# Characteristics and risk factors of secondary bacterial infections in COVID-19 patients

**DOI:** 10.1017/ash.2023.425

**Published:** 2023-09-13

**Authors:** Guangjie Wu, Jianhua Lu, Dong Liu, Yan He

**Affiliations:** 1 Department of Pharmacy, Tongji Hospital, Tongji Medical College, Huazhong University of Science and Technology, Wuhan, Hubei Province, China; 2 Department of Information, ZhuJiang Hospital, Southern Medical University, Guangzhou, Guangdong Province, China

**Keywords:** COVID-19, secondary bacterial infections, risk factor, antibiotic

## Abstract

**Objective:**

To describe the characteristics and find out risk factors of COVID-19 patients infected with different categories of bacteria.

**Design:**

Case-control.

**Methods:**

We conducted a retrospective study including 129 COVID-19 patients admitted to a tertiary hospital between October 13, 2022 and December 31, 2022. Patients’ data were collected from the hospital information system. Patients were classified as having or not having confirmed secondary bacterial infections, or gram-positive and gram-negative bacterial infections for analysis. Categories and sources of isolated bacteria, characteristics of the patients, and the risk factors for developing secondary bacterial infections were analyzed.

**Results:**

Gram-negative bacteria accounted for the majority of secondary bacterial infections of the included patients. Critical type of COVID-19 (OR = 12.98, 95%CI 3.43∼49.18, *p* < 0.001), invasive therapy (OR = 9.96, 95%CI 3.01∼32.95, *p* < 0.001), and previous antibiotics use (OR = 17.23, 95%CI 1.38∼215.69, *p* = 0.027) were independent risk factors of secondary bacterial infections in COVID-19 patients. Ceftriaxone/cefotaxime use (OR = 15.45, 95%CI 2.72∼87.79, *p* = 0.002) was associated with gram-positive bacterial infections while age over 70 (OR = 3.30, 95%CI 1.06∼10.26, *p* = 0.039), invasive therapy (OR = 4.68, 95%CI 1.22∼17.93, *p* = 0.024), and carbapenems use (OR = 8.48, 95%CI 2.17∼33.15, *p* = 0.002) were associated with gram-negative bacterial infections.

**Conclusions:**

Critical patients with invasive therapy and previous antibiotics use should be cautious with secondary bacterial infections. Third-generation cephalosporins and carbapenems should be used carefully because both are risk factors for gram-positive or gram-negative bacterial infections.

## Introduction

The COVID-19 pandemic continues to constitute a public health issue, as pronounced by the World Health Organization.^1^ Patients with viral infections are predisposed to secondary bacterial infections, which are defined as infections occurred more than 48 hours after hospitalization.^2^ Recent meta-analysis showed that the prevalence rate of secondary bacterial infections was 18.4%.^3^ However, antibiotics were still empirically prescribed for 62% of COVID-19 patients, which was far surpassing the rate of bacterial infections.^2^ The decision to use antibiotics for patients with COVID-19 is often considered as a prophylactic therapy in order to prevent potential secondary bacterial infections. However, there is currently insufficient evidence supporting such prophylactic use of antibiotics.^4^ On the other hand, distinguishing between viral and bacterial infections, especially for patients with severe and critical conditions, can be challenging due to similarities in chest images, false negative or false positive results in laboratory tests, and time lags in microbial culture.^5^ These factors may contribute to the overuse and misuse of antibiotics for COVID-19 patients.

High probability of antibiotic use during COVID-19 pandemic prompts concerns about selection of antibiotic-resistant bacteria. A retrospective study found that multi-drug-resistant pathogens were only detected in the antibiotic-exposed group of COVID-19 patients,^6^ and antibiotic exposure was an independent risk factor for development of antibiotic-resistant bacterial secondary infection.^6,7^ It can be concluded that irrational antibiotic use could lead to worse outcomes in COVID-19 patients.^8^ Thus, only by administering the correct antibiotics to COVID-19 patients with the risk of secondary bacterial infections could benefits be obtained.

To find out the risk factors of bacterial infections in COVID-19 patients is important. So far, risk factors of bacterial infection in COVID-19 have been studied in several articles. Male sex, diabetes, hematological disease, mechanical ventilation, invasive devices, combination of antibiotics, and glucocorticoid treatment were found to be related to secondary bacterial infections in different articles.^9–11^ However, knowing only the risk factors of secondary bacterial infection seems not enough, because this would result in broad-coverage of potential bacteria in COVID-19 patients with meropenem, piperacillin/tazolbactam, and ceftriaxone.^12^ Unfortunately, broad-spectrum antibiotic use contributed to a rapid spread of drug-resistant bacteria,^13^ which has no good for the prognosis of COVID-19 patients. So it is still needed to understand whether different conditions can lead to secondary infections caused by different bacteria in COVID-19 patients for the purpose of using antibiotics with a focused target.

Based on these facts, we plan to further analyze risk factors associated with secondary infections caused by different bacteria of COVID-19 patients, in order to provide more information on better antibiotic use with COVID-19 patients.

## Methods and materials

We retrospectively reviewed patients admitted to a tertiary hospital in Wuhan, Hubei Province, China between October 1, 2020 and December 31, 2022. The inclusion and exclusion criteria were as follows:

Inclusion criteria: (1) with confirmed diagnosis of COVID-19 by polymerase chain reaction testing before admission and (2) with clear bacterial cultural results before discharge. Exclusion criteria: (1) positive bacterial cultural results from applications <48 hours after admission (2) with only one positive bacterial cultural result that was suspicious of contamination (3) with records of clear diagnosis of bacterial infections before admission. Finally, 129 patients were included, and we performed a 1:2 case-control study with 43 patients with confirmed secondary bacterial infections assigned to the case group and 86 assigned to the control group.

Data on baseline characteristics were collected from the hospital information system. Therapy records and medication records only before the (first positive) microbial culture application were included for analysis. Therapy was classified into noninvasive and invasive procedures. The definition of invasive procedure was previously described^14^ and included invasive ventilators (tracheal intubation, tracheotomy), vascular devices (peripherally inserted central venous catheters, central venous catheter), renal replacement therapy, and indwelling urinary catheters.

### Statistical analysis

Categorical variables were presented as frequencies with percentages and Chi-square method was used to test the significance. Kolmogorov–Smirnov test and Shapiro–Wilk test were used to test the normality of continuous variables according to the sample size (for sample >50 and sample ≤50, using K-S test and S–W test, respectively). Levene test was used to detect the homogeneity of variance. Continuous variables were then presented as mean±SD (standard deviation) or medians with interquartile range based on the normality. T-test was used for significance test of continuous variables, while Mann–Whitney U-test was used when the criteria of T-test was not satisfied. To assess the association between risk factors and bacterial infections, multivariate logistic regression models were used, and factors included in the model were based on the results of univariate significance test. All analyses were considered significant at two-tailed *p*-values of <0.05.

## Results

### Species and sources of the microbial findings

The average time between the first positive bacterial cultural result and the occurrence of first COVID-19 symptoms as well as the admission time were 32.07 ± 17.32 days and 18.95 ± 12.66 days, respectively (data were not shown). In total, 57 strains of bacteria were found in the 43 patients as described in Table 1. Acinetobacter baumannii, Klebsiella pneumoniae, Escherichia coli, Enterococcus faecium, MRSA, and Stenotrophomonas maltophilia ranked the first 6 of the isolated bacteria. Of the 57 strains, gram-negative bacteria accounted for the majority. The sources of the bacteria were 20 from sputum, 18 from blood, 15 from urine, and 4 from BAL, as described in Table 1.

**Table 1.** Categories and species of isolated bacteria from the 43 COVID-19 patients^a^

Category	Species	Number (%)	Sources/Numbers			
			Sputum	Blood	Urine	BAL
GPC	Enterococcus faecium	6 (10.53)	0	3	3	0
	MRSA	5 (8.77)	2	3	0	0
	MRSE	1 (1.75)	1	0	0	0
	Micrococcaceae Pribram	1 (1.75)	1	0	0	0
GPB	Corynebacterium striatum	1 (1.75)	1	0	0	0
GNB	Acinetobacter baumannii	10 (17.54)	3	5	1	1
	Klebsiella pneumoniae	9 (15.79)	3	4	2	0
	Escherichia coli	9 (15.79)	0	2	7	0
	Stenotrophomonas maltophilia	5 (8.77)	4	0	0	1
	Pseudomonas aeruginosa	3 (5.26)	2	0	0	1
	Proteus mirabilis	2 (3.51)	1	0	1	0
	Proteus vulgaris	1 (1.75)	0	0	0	1
	Enterobacter aldermani	1 (1.75)	0	1	0	0
	Klebsiella oxytoca	1 (1.75)	0	0	1	0
	Ralstonia	1 (1.75)	1	0	0	0
	Serratia marcescens	1 (1.75)	1	0	0	0

a This table shows the species and sources of isolated bacteria in COVID-19 patients.

Note. GPC, gram-positive cocci; GPB, gram-positive bacilli; GNB, gram-negative bacilli; MRSA, methicillin-resistant Staphylococcus aureus; MRSE, methicillin- resistant Staphylococcus epidermidis. BAL, bronchoalveolar lavage.

### Laboratory findings of COVID-19 patients with confirmed secondary bacterial infections

In COVID-19, patients with confirmed bacterial infections, blood level of neutrophils, CRP, ferritin, IL-1β, IL-2R, IL-6, and TNF-α were higher than the upper limits of the normal values with significant differences, while lymphocyte was significantly lower. Though not statistically significant, WBC, IL-8, and IL-10 seemed higher in the bacterial-infected patients, too. The median value of procalcitonin (PCT) was slightly lower than the upper limit of the normal value, but no statistical difference was found. Details are shown in Table 2.

**Table 2.** Laboratory findings of COVID-19 patients with confirmed secondary bacterial infections^a^

Indicators	Mean ± SD	Median (Inter-quartile range)	Reference value (Upper limit)	*p*
WBC (×10^9^/L)	10.79 ± 6.00	–	9.50	0.167
N (×10^9^/L)	9.15 ± 6.00	–	6.30	0.003*
L (×10^9^/L)	–	0.66 (0.44, 0.78)	3.20	0.000*
PCT (ng/mL)	–	0.31 (0.08, 2.28)	0.50	0.422
CRP (mg/L)	–	61.70 (23.20, 181.40)	1.00	0.000*
Ferritin (μg/L)	–	1206.10 (676.20, 2361.0)	400.00	0.000*
IL-1β (pg/mL)	–	5.60 (5.00, 11.60)	5.00	0.000*
IL-2R (U/mL)	–	1121.00 (658.00, 2427.00)	710.00	0.001*
IL-6 (pg/mL)	–	77.79 (30.49, 541.80)	7.00	0.000*
IL-8 (pg/mL)	–	65.7 (21.90, 1538.00)	62.00	0.464
IL-10 (pg/mL)	–	10.50 (5.60, 46.80)	9.10	0.067
TNF-α (pg/mL)	–	13.90 (9.80, 41.40)	8.10	0.000*

a This table shows characteristics of laboratory findings of COVID-19 patients with confirmed secondary bacterial infections. The reference values of each indicator are based on the standards in our laboratory.

Note. WBC, white blood cell counts; N, neutrophils; L, lymphocytes; PCT, procalcitonin; CRP, C-reaction protein; IL, interleukins; IL-2R, interleukin-2 receptors; TNF-α, tumor necrosis factor-α.

### Overall and subgroup analysis on characteristics of the patients with or without bacterial infections

Though not significant, patients with bacterial infections seemed older than those without a confirmed bacterial infection (mean age 68.81 ± 11.32 vs 65.77 ± 10.94). There were no significant differences in sex, smoking history, and comorbidities between the two groups. Clinical type of COVID-19 showed statistical differences in subgroup analysis of total bacterial infections, gram-positive bacterial infections, and gram-negative bacterial infections, with a higher proportion of critical type in the infection group. As for therapy and medication, invasive ventilators, invasive vascular devices, renal replacement therapy, indwelling urinary catheters, antibiotics, and glucocorticoids were found with significant differences, which were more common in the infection group. There was also a significant difference in the median duration of glucocorticoid between the two groups (4 vs 0). Treating with noninvasive ventilators and using tocilizumab were not significantly different when comparing the two groups. For specific antibiotics, treating with ceftriaxone/cefotaxime, ceftazidime/cefoperazone sulbactam, carbapenems, glycopeptides, and linezolid were more common in patients with secondary bacterial infections, while differences of other antibiotics were not statistically significant.

For COVID-19 patients infected with gram-positive bacteria, demographic characteristics of age, sex, smoking history, and comorbidities were found with no significance. Compared with using noninvasive ventilators, invasive ventilators were more common in the infected patients. Besides, there were significant differences in invasive vascular devices, and indwelling urinary catheter use between the two groups, which had larger proportions in the infected patients. However, renal replacement therapy was found without significant difference when comparing the two groups. For medication, glucocorticoid, overall antibiotics use as well as tocilizumab were all found with no significant differences between the two groups, though patients with confirmed bacterial infections had higher frequencies of using these three medications. In terms of antibiotic categories, third-generation cephalosporins including ceftriaxone, cefotaxime, ceftazidime, and cefoperazone sulbactam, as well as carbapenems, were found more frequent in the infected patients, while the other antibiotics were found not statistically different between the two groups.

On the other hand, characteristics of patients infected with or without gram-negative bacteria varied. Ages were significantly higher in patients infected with gram-negative bacteria when compared with those without confirmed gram-negative bacteria infections. The rest demographic characteristics, including sex, smoking history, and comorbidities, were not significantly different between the two groups. Similarly, more invasive ventilators, invasive vascular devices, and urinary catheters were found in patients infected with gram-negative bacteria than those without infection of such bacteria. However, frequencies of renal replacement therapy were not significantly different between the two groups. For medications, overall antibiotic use and using tocilizumab were also found with no differences between the two groups. Differently, using glucocorticoid was found more frequently in patients with bacterial infections and the median duration of glucocorticoid was higher (3.5 vs 0) in the case group. Also, in terms of antibiotic categories, carbapenems and glycopeptides were more common in patients with gram-negative bacterial infections, which was different from the results between patients with and without gram-positive bacterial infections. Details are shown in Table 3.

**Table 3.** Subgroup analysis on characteristics of patients based on bacteria categories^a^

Variables	Overall secondary infections			Gram-positive bacteria			Gram-negative bacteria		
	Without infection	With infection	*p*	Without infection	With infection	*p*	Without infection	With infection	*p*
	(*n* = 86)	(*n* = 43)		(*n* = 115)	(*n* = 14)		(*n* = 97)	(*n* = 32)	
**Demographic**									
Age	.65.77 ± 10.94	68.81 ± 11.32	0.143	67.13 ± 10.98	63.93 ± 11.24	0.311	65.22 ± 10.89	71.53 ± 10.58	0.005*
Sex, female	37 (43.0)	22 (51.2)	0.382	51 (44.3)	8 (57.1)	0.364	43 (44.3)	16 (50.0)	0.577
Smoking history	10 (11.6)	8 (18.6)	0.281	15 (13.0)	3 (21.4)	0.655	13 (13.4)	5 (15.6)	0.984
**Clinical type**									
Severe	77 (89.5)	10 (23.3)	0.000*	85 (73.9)	2 (14.3)	0.000*	78 (80.4)	9 (28.1)	0.000*
Critical	9 (10.5)	33 (76.7)		30 (26.1)	12 (85.7)		19 (19.6)	23 (71.9)	
**Comorbidities**	60 (69.8)	32 (74.4)	0.582	82 (71.3)	10 (71.4)	1.000	67 (69.1)	25 (78.1)	0.326
Hypertension	45 (52.3)	20 (46.5)	0.534	57 (49.6)	8 (57.1)	0.592	50 (51.5)	15 (46.9)	0.647
Diabetes Mellitus	20 (23.3)	8 (18.6)	0.546	26 (22.6)	2 (14.3)	0.711	22 (22.7)	6 (18.8)	0.640
Pulmonary disease	14 (16.3)	9 (20.9)	0.515	21 (18.3)	2 (14.3)	1.000	15 (15.5)	8 (25.0)	0.222
Coronary heart disease	16 (18.6)	6 (14.0)	0.508	21 (18.3)	1 (7.1)	0.504	16 (16.5)	6 (18.8)	0.769
Renal dysfunction	3 (5.3)	4 (9.3)	0.336	5 (4.3)	2 (14.3)	0.168	5 (5.2)	2 (6.3)	1.000
Hematopathy	2 (2.3)	1 (2.3)	1.000	3 (2.6)	0 (0.0)	1.000	2 (2.1)	1 (3.1)	1.000
Tumor	2 (3.5)	1 (2.3)	1.000	3 (3.5)	0 (0.0)	1.000	2 (2.1)	1 (3.1)	1.000
**Therapy and medication**									
Noninvasive ventilator	80 (93.0)	37 (86.0)	0.335	106 (92.2)	11 (78.6)	0.243	89 (91.8)	28 (87.5)	0.713
**Invasive therapy**	18 (20.9)	37 (86.0)	0.000*	42 (36.5)	13 (92.9)	0.000*	28 (28.9)	27 (84.4)	0.000*
Invasive ventilator	5 (5.8)	28 (65.1)	0.000*	21 (18.3)	12 (85.7)	0.000*	15 (15.5)	18 (56.3)	0.000*
Invasive vascular device	3 (3.5)	29 (67.4)	0.000*	21 (18.3)	11 (78.6)	0.000*	12 (12.4)	20 (62.5)	0.000*
Renal replacement therapy	4 (4.7)	11 (25.6)	0.000*	11 (9.6)	4 (28.6)	0.098	8 (8.2)	7 (21.9)	0.077
Indwelling urinary catheter	17 (19.8)	35 (81.4)	0.000*	40 (34.8)	12 (85.7)	0.000*	26 (26.8)	26 (81.3)	0.000*
Using antibiotics	67 (77.9)	42 (97.7)	0.003*	95 (82.6)	14 (100.0)	0.191	78 (80.4)	31 (96.9)	0.051
Using glucocorticoid	21 (24.4)	26 (60.5)	0.000*	39 (33.9)	8 (57.1)	0.088	29 (29.9)	18 (56.3)	0.007*
Duration of glucocorticoid	0 (0, 1)	4 (0, 7)	0.000*	0 (0, 4.00)	4 (0, 5.50)	0.121	0 (0, 3.50)	3.5 (0, 7.00)	0.002*
Using tocilizumab	1 (1.2)	3 (7.0)	0.209	2 (1.7)	2 (14.3)	0.058	3 (3.1)	1 (3.1)	1.000
**Antibiotics categories**									
Ceftriaxone/Cefotaxime	9 (10.5)	12 (27.9)	0.011*	13 (11.3)	8 (57.1)	0.000*	15 (15.5)	6 (18.8)	0.662
Ceftazidime/Cefoperazone sulbactam	19 (22.1)	24 (55.8)	0.000*	33 (28.7)	10 (71.4)	0.004*	28 (28.9)	15 (46.9)	0.061
Penicillins (with β-lactamase inhibitors)	4 (4.7)	7 (16.3)	0.058	8 (7.0)	3 (21.4)	0.186	7 (7.2)	4 (12.5)	0.573
Carbapenems	5 (5.8)	30 (69.8)	0.000*	26 (22.6)	9 (64.3)	0.003*	12 (12.4)	23 (71.9)	0.000*
Glycopeptides	1 (1.2)	9 (20.9)	0.000*	9 (7.8)	1 (7.1)	1.000	2 (2.1)	8 (25.0)	0.000*
Fluoroquinolones	61 (70.9)	29 (67.4)	0.684	80 (69.6)	10 (71.4)	1.000	70 (72.2)	20 (62.5)	0.302
Aminoglycosides	0 (0.0)	2 (4.7)	0.109	1 (0.9)	1 (7.1)	0.206	1 (1.0)	1 (3.1)	0.436
Linezolid	0 (0.0)	6 (14.0)	0.002*	4 (3.5)	2 (14.3)	0.128	2 (2.1)	4 (12.5)	0.051
Ceftazidime avibactam	1 (1.2)	1 (2.3)	1.000	2 (1.7)	0 (0.0)	1.000	1 (1.0)	1 (3.1)	0.436
Sulfamethoxazole Co.	1 (1.2)	4 (9.3)	0.076	4 (3.5)	1 (7.1)	0.442	2 (2.1)	3 (9.4)	0.183
Daptomycin	0 (0.0)	2 (4.7)	0.109	1 (0.9)	1 (7.1)	0.206	1 (1.0)	1 (3.1)	0.436

a This table shows demographic information, clinical types, comorbidities, therapy, and medications as well as specific antibiotics of COVID-19 patients with or without secondary bacterial infections or infected with or without specific bacterial categories.

### Risk factors of overall, gram-positive, and gram-negative bacterial secondary infections

**Figure 1.**
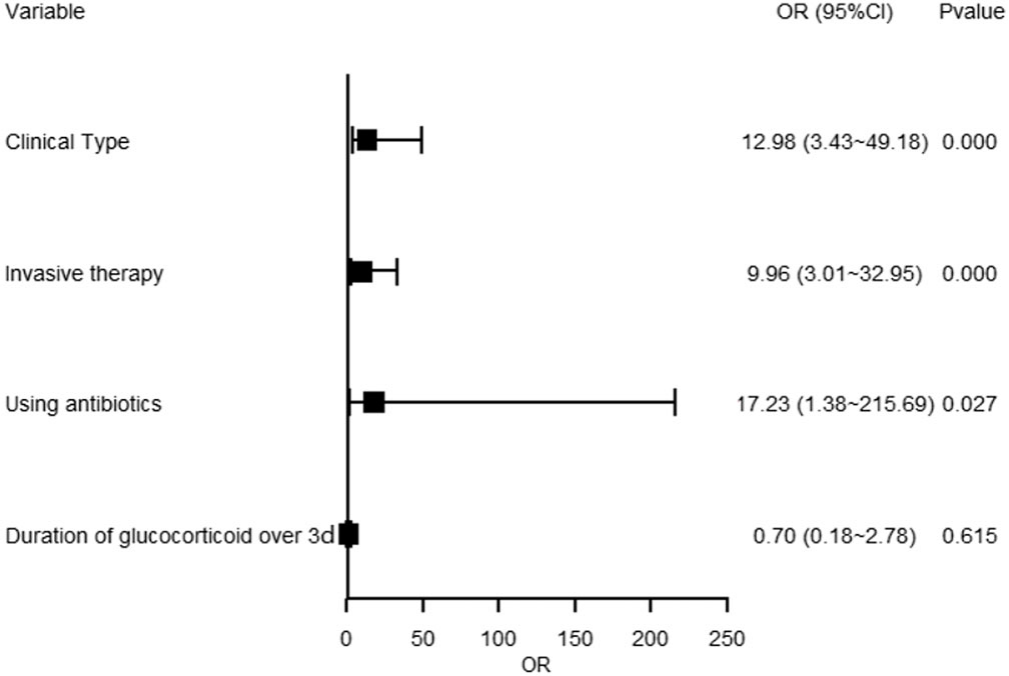
Forest plot showing multivariate regression model of overall secondary bacterial infections. Blocks represented the OR values between the case group and the control group. Lines represented 95% confidence interval.

For overall secondary bacterial infections, clinical type of COVID-19, invasive therapy, using antibiotics, and duration of glucocorticoid over 3 days were included in the regression model. Critical type was associated with overall secondary bacterial infections (OR = 12.98, 95%CI 3.43∼49.18, *p* < 0.001). Invasive therapy was also found as a risk factor for overall bacterial infections (OR = 9.96, 95%CI 3.01∼32.95, *p* < 0.001). Similarly, using antibiotics was associated with overall bacterial infections (OR = 17.23, 95%CI 1.38∼215.69, *p* = 0.027), while duration of glucocorticoid over 3 days was not (OR = 0.70, 95%CI 0.18∼2.78, *p* = 0.615). See Figure 1.

**Figure 2.**
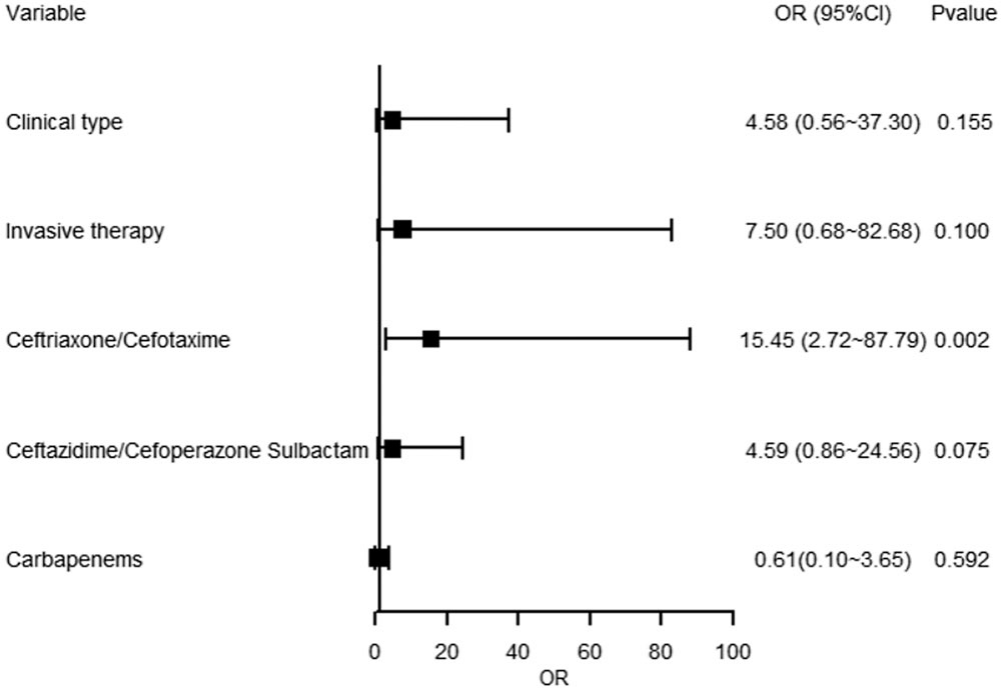
Forest plot showing multivariate regression model of gram-positive bacterial infections in COVID-19 patients. Blocks represented the OR values between the case group (gram-positive) and the control group. Lines represented 95% confidence interval.

For gram-positive bacterial infections, clinical type and invasive therapy were not found with significant association with the infections (*p* = 0.155 and *p* = 0.100, respectively). In terms of antibiotics categories, using ceftriaxone/cefotaxime was associated with developing gram-positive bacterial infections (OR = 15.45, 95%CI 2.72∼87.79, *p* = 0.002), while using ceftazidime/cefoperazone sulbactam and carbapenems were not (*p* = 0.075 and *p* = 0.592, respectively). See Figure 2.

**Figure 3.**
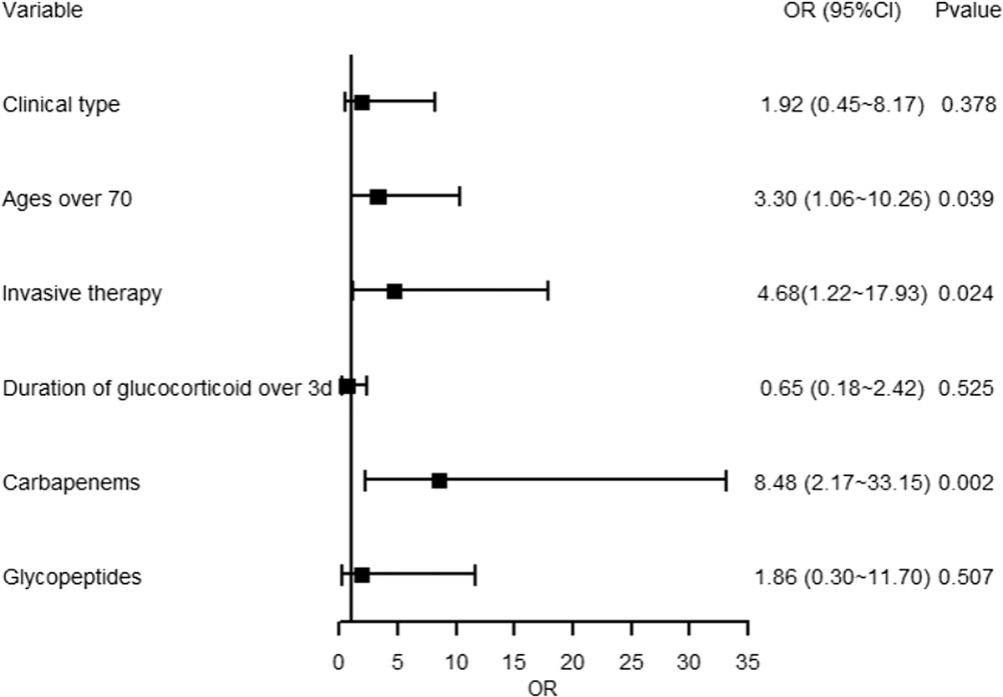
Forest plot showing multivariate regression model of gram-negative bacterial infections in COVID-19 patients. Blocks represented the OR values between the case group (gram-negative) and the control group. Lines represented 95% confidence interval.

For gram-negative bacterial infections, we found that patients with ages over 70 were at a risk of developing gram-negative bacterial infection (OR = 3.30, 95%CI 1.06∼10.26, *p* = 0.039). Likely, invasive therapy was also associated with gram-negative bacterial infections (OR = 4.68, 95%CI 1.22∼17.93 *p* = 0.024). In terms of medications, using carbapenems was a risk factor of the infections (OR = 0.002, 95%CI 2.17∼33.15, *p* = 0.002), while clinical type, glucocorticoid, and glycopeptides were not independent risk factors in the regression model. See Figure 3.

## Discussion

Secondary bacterial infections are important causes of morbidity and mortality in severe viral infectious diseases including COVID-19. Understanding its characteristics is of great significance for treatment initiation and scheme determination. In a large systematic review on microbiology of respiratory and bloodstream bacterial infections in COVID-19 patients, Bradley et al included 171 studies with 171 262 patients and found that the proportions of Staphylococcus, Acinetobacter, Escherichia, Klebsiella, Pseudomonas, and Enterococcus accounted for the most.^11^ The bacterial results in our study were similar. The main difference we found was the higher detection rate of Stenotrophomonas maltophilia. Meta-analysis has concluded the risk factors associated with Stenotrophomonas maltophilia infections, such as severity of conditions, comorbidities, and mechanical ventilation as well as specific antibiotics.^15^ In a matched case-control study, previous ureido/carboxypenicillin and carbapenem use were found as independent risk factors of Stenotrophomonas maltophilia ventilation-acquired pneumonia.^16^ This could explain the higher detection rate of the pathogen in our study since the five patients with Stenotrophomonas maltophilia infections were all previously treated with carbapenems.

Laboratory findings are often used for aiding in diagnosis of bacterial infections and as a guide to initiate antibiotic therapy. In our study, we found that most inflammatory indicators increased, which was consistent with other studies.^5,7,17^ Unlike most studies, PCT of COVID-19 patients with confirmed bacterial infections in our study did not increase to a breakpoint at which bacterial infections were commonly considered. PCT is a peptide released in response to pro-inflammatory stimuli, especially bacterial infections, and has been useful as a diagnostic indicator to discriminate between bacterial and viral infections.^18^ As reported by Huang et al, 75% COVID-19 patients had elevated PCT > 0.5 ng/mL.^19^ In fact, PCT has been used widely in COVID-19 literature^20^ and is considered a useful tool for reducing antibiotics usage. We might explain the unusual finding for several reasons. Firstly, the cut-off value of PCT was uncertain for bacterial infections. In a systematic review of PCT use in COVID-19 patients for detecting bacterial infections, Meier et al described different PCT cut-off values in the published papers and found that the range was very broad, from 0.1 to 0.5 ng/mL.^21^ It should be noted that we used the upper limit (0.5 ng/mL) of serum PCT from the hospital laboratory as the comparison instead of statistical results of COVID-19 patients without bacterial infections. The reason was that we were unable to determine at which time point the laboratory test results could be used as a reference in the control group. Secondly, it was reported that serum interferon-gamma synthesis increase could inhibit PCT secretion, especially in respiratory viral infections.^22^ As COVID-19 is a viral disease, we could reasonably explain the relatively low PCT levels in our study based on this result. But we still must acknowledge that more data on PCT of COVID-19 patients with confirmed bacterial infections are needed. In terms of other biomarkers, lymphopenia was found in the bacterial infectious group, which was within up to 85% of severe COVID-19 patients and the decrease in lymphocytes could be explained by more severe conditions of patients who were with secondary bacterial infections.^23^ We also found an increase in neutrophils. Neutrophilia was associated with high-inflammatory status and cytokine storm, and the latter is part of the pathogenesis of COVID-19, which is common in patients with severe COVID-19.^24^ Of course, the increase in neutrophils could also be caused by secondary bacterial infection. Collectively, when neutrophils are elevated in patients with COVID-19, poor outcomes and possible bacterial infections should be considered. As for cytokines, IL-1β, IL-2R, IL-6, and TNF-α were found with significant increases in patients with confirmed secondary infections, which was consistent with Lee’s research.^25^ Interestingly, there were differences in cytokine changes between COVID-19 and other viral respiratory infections such as influenza. It was reported that responses to interferon I/II pathways were more associated with the influenza whereas pathways for the response to TNF-α or IL-1β were more prominent in COVID-19,^25^ besides IL-1β was also amongst the identified strongest markers of ventilator-associated pneumonia,^26^ and our results aligned with these conclusions.

It is always interesting to understand the risk factors of secondary bacterial infections in COVID-19 patients, and it is also with great significance of antibiotic rational use for physicians and pharmacists since reports have confirmed that secondary bacterial infections were a dependent factor of in-hospital mortality in COVID-19 patients.^5,7^ In our study, we identified critical COVID-19 type, previous antibiotics use, and invasive therapy were the three risk factors of overall bacterial infections. In a retrospective study of 201 COVID-19 patients, Iacovelli et al found exposure to antibiotic therapy in last 30 days (OR = 4.82, 95%CI1.28∼18.1, *p* = 0.020) was an independent risk factor for new-onset superinfections development,^7^ and this result was consistent with ours. Invasive therapies including invasive ventilators, blood devices, and urinary catheters were often used in critical COVID-19 patients. Retrospective studies have shown that in COVID-19 patients, especially those who were admitted into ICUs, longer duration of invasive mechanical ventilation, blood devices, and indwelling urinary catheters were associated with higher bacterial superinfection rates.^22,27^ These results along with ours make two suggestions, the first is that strict antibiotic stewardship should be carried out for COVID-19 patients to reduce the risks of secondary bacterial infections, and the second is that for those patients who need invasive therapies, secondary bacterial infections must be cautious with.

In the subgroup analysis based on bacterial species, we found specific antibiotic categories were risk factors for different bacterial infections. For gram-positive bacteria, ceftriaxone/cefotaxime increased the infection rates. This could be explained as a selection of microbiome with disruption of colonization and proliferation of gram-positive bacteria since majority isolation were Enterococcus faecium and MRSA, which were not sensitive to third-generation cephalosporins.^28^ In vitro experiments have described this mechanism. It was shown that ceftriaxone application with mice led to a reduction of the mucus-associated microbiota layer and segregation of Enterococcus faecium from the intestinal wall, thus leading to more frequent infection of the bacteria.^29,30^ As for gram-negative bacterial infections, we found previous carbapenems use was an independent risk factor for these infections. The result was also consistent with other studies. In a meta-analysis, Karlijn et al showed that carbapenems use increased the risk with a fold of 4.71 as the second factor of carbapenem-resistant Enterobacteriaceae infections.^31^ In another systematic review by Zaira et al,^32^ the authors analyzed 92 articles and found that 82.6% of the studies reported previous carbapenems use was associated with multiple-drug-resistant (MDR) gram-negative bacterial infections. Since most of the isolated gram-negative bacteria in our COVID-19 samples were MDR bacteria (data were not shown), we could see from these results that in COVID-19 patients, carbapenems should be carefully used since this kind of antibiotic was an independent risk factor for infections caused by gram-negative bacteria, especially MDR or carbapenem-resistant species.

Limitations existed in this study. First, this was a single-center study, and the sample size of patients with gram-positive bacterial infections was relatively small, so we were not sure whether this would affect the results of gram-positive bacterial infections. Second, it was common for physicians to give antibiotics to patients under severe or critical conditions even without very clear evidence of bacterial infections. We included some patients who had previously received antibiotics and were classified as the control because they did not have a clear diagnosis of bacterial infection and did not have positive bacterial culture results after admission, and this probably brought some bias. More work is still needed.

In summary, in this retrospective study, we’ve described the microbiology and clinical characteristics of COVID-19 patients with confirmed secondary bacterial infections. Critical patients with invasive therapy and previous antibiotics use should be cautious with secondary bacterial infections. Third-generation cephalosporins and carbapenems should be used carefully because both are risk factors for gram-positive or gram-negative bacterial infections.
